# Supplementary material for: Moderate Soil Drying-Induced Alternative Splicing Provides a Potential Novel Approach for the Regulation of Grain Filling in Rice Inferior Spikelets
Source: Int J Mol Sci. 2022 Jul 14;23(14):7770. doi: 10.3390/ijms23147770 (PMC9318316; doi:10.3390/ijms23147770)
Supplement: Supplementary file 1 [file ijms-23-07770-s001.zip › Supplemental Figures and Table.pdf]

Moderate Soil Drying-Induced Alternative Splicing Provides a Potential Novel Approach for the Regulation of Grain Filling in Rice Inferior Spikelets

Zhenning Teng [1,2,†], Qin Zheng [1,†], Bohan Liu [1,3], Shuan Meng [1,3], Jianhua Zhang [4,*] and Nenghui Ye [1,3,*]

1 College of Agronomy, Hunan Agricultural University, Changsha 410128, China; sailingtzn@163.com (Z.T.); zhengqin@stu.hunau.edu.cn (Q.Z.); liubohan@hunau.edu.cn (B.L.); mengshuanz@outlook.com (S.M.)
2 Shenzhen Research Institute, The Chinese University of Hong Kong, Shenzhen 518057, China
3 Key Laboratory of Crop Physiology and Molecular Biology, Ministry of Education,
Hunan Agricultural University, Changsha 410128, China
4 Department of Biology, Hong Kong Baptist University, Kowloon, Hong Kong 999077, China
* Correspondence: jzhang@hkbu.edu.hk (J.Z.); nye@hunau.edu.cn (N.Y.)
† These authors contributed equally to this work.

Supplementary Figure Legends

Supplementary Figure S1 Alternative splicing events

Supplementary Figure S2 DAS genes involved in spliceosome by KEGG analysis in inferior spikelet at 9 DAA under CK and MD treatment.

Supplementary Figure S3 DAS genes involved in starch and sucrose metabolism by KEGG analysis in inferior spikelet at 9 DAA under CK and MD treatment.

Supplementary Figure S4 Small-RNA sequencing result of osa-miR444b and osa-miR439f

Supplementary Table Legends

Supplementary Table S1 Summary of AS type of rice inferior spikelets under CK and MD conditions

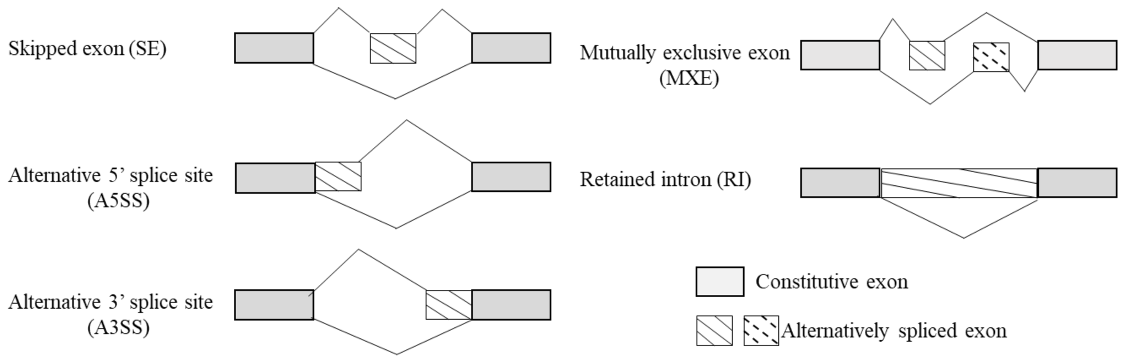

Figure S1. Alternative splicing events

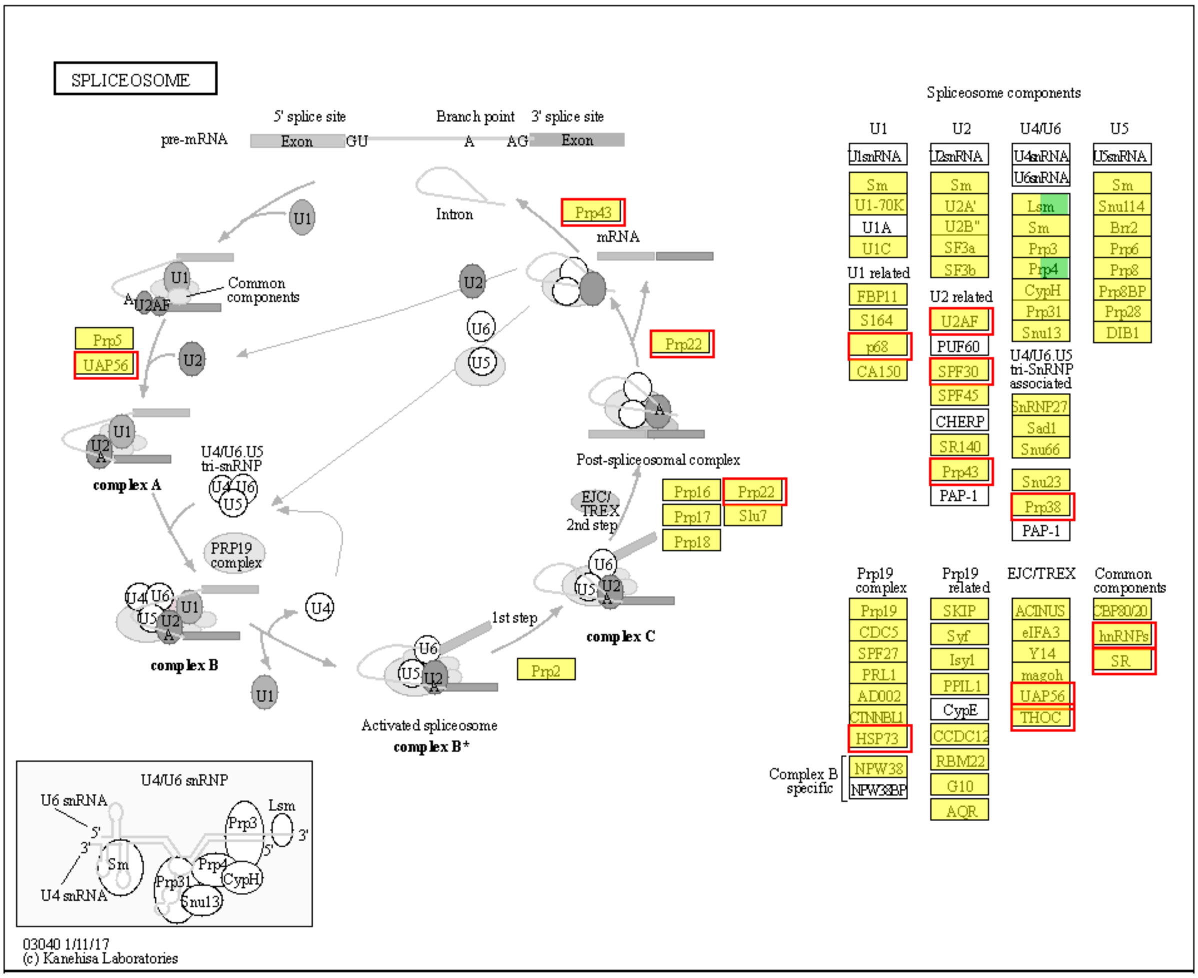

Figure S2. DAS genes involved in spliceosome by KEGG analysis in inferior spikelet at 9 DAA under CK and MD treatment.

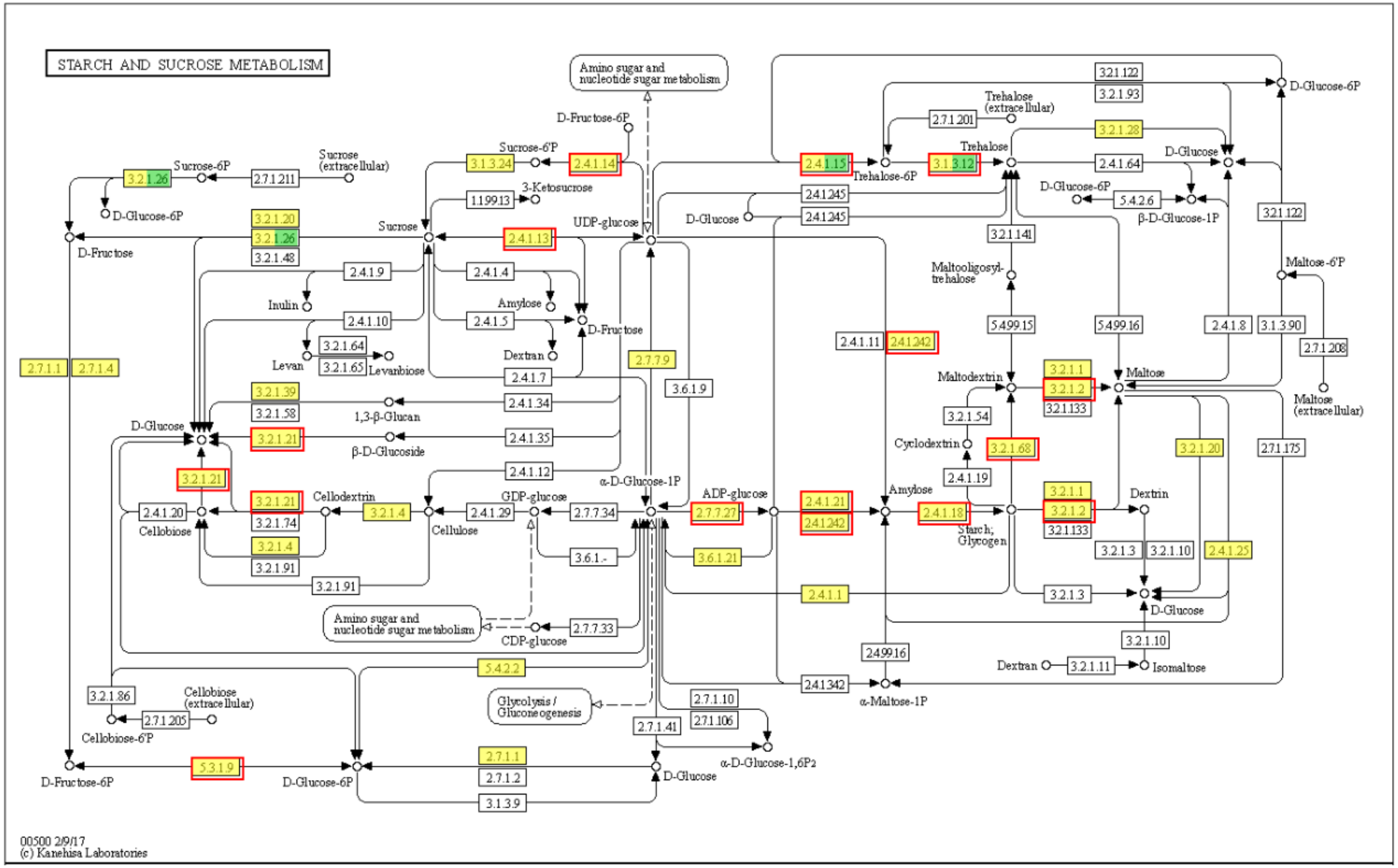

Figure S3. DAS genes involved in starch and sucrose metabolism by KEGG analysis in inferior spikelet at 9 DAA under CK and MD treatment.

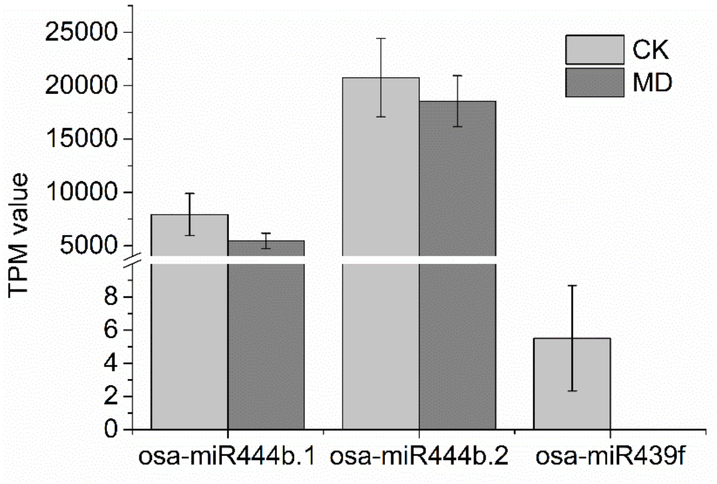

Figure S4. Small-RNA sequencing result of osa-miR444b and osa-miR439f

Table S1. Summary of AS type of rice inferior spikelets under CK and MD conditions

Treatment	SE	RI	A5SS	A3SS	MXE	Total
CK	6548±18	2457±66	1747±32	3166±33	347±5	14264±146
MD	6724±35 **	3288±41 **	1964±19 **	3450±38 **	362±2 **	15788±131 **
